# Prioritization strategies for pandemic influenza vaccine in 27 countries of the European Union and the Global Health Security Action Group: a review

**DOI:** 10.1186/1471-2458-7-236

**Published:** 2007-09-07

**Authors:** Masja Straetemans, Udo Buchholz, Sabine Reiter, Walter Haas, Gérard Krause

**Affiliations:** 1 Department of Infectious Disease Epidemiology, Robert Koch-Institut, Seestrasse 10, 13352 Berlin, Germany

## Abstract

**Background:**

Although there is rapid progress in vaccine research regarding influenza pandemic vaccines it is expected that pandemic influenza vaccine production can only start once the pandemic virus has been recognized. Therefore, pandemic vaccine capacity will be limited at least during the first phase of an influenza pandemic, requiring vaccine prioritization strategies. WHO recommends developing preliminary priorities for pandemic vaccine use. The goal of this review is to provide a thorough overview of pandemic vaccine prioritization concepts in the 27 European Union (EU) member states and the four non-EU countries of the Global Health Security Action Group.

**Methods:**

Between September and December 2006 data was collected for each country through two data sources: (i) the national influenza pandemic plan; (ii) contacting key persons involved in pandemic planning by email and/or phone and/or fax

**Results:**

Twenty-six (84%) countries had established at least one vaccine priority group. Most common reported vaccine priority groups were health care workers (HCW) (100%), essential service providers (ESP) (92%) and high risk individuals (HRI) (92%). Ranking of at least one vaccine priority group was done by 17 (65%) of 26 countries. Fifteen (88%) of these 17 countries including a ranking strategy, decided that HCW with close contact to influenza patients should be vaccinated first; in most countries followed and/or ranked equally by ESP and subsequently HRI. Rationales for prioritization were provided by 22 (85%) of 26 countries that established vaccine priority groups. There was large variation in the phrasing and level of detailed specification of rationales. Seven (32%) of 22 countries providing rationales clearly associated each vaccine priority group with the specific rationale. Ten (32% of the 31 countries studied) countries have consulted and involved ethical experts to guide decisions related to vaccine prioritization.

**Conclusion:**

In the majority of the countries the establishment of vaccine priority groups, ranking and underlying rationales are in line with WHO recommendations. In most public plans the criteria by which prioritized groups are identified are not easily recognizable. Clarity however, may be necessary to assure public acceptability of the prioritization. Ethical experts, results of modelling exercises could play an increasing role in the future decision making process.

## Background

In 2005, WHO has strongly recommended to its member states to develop or update their national influenza pandemic preparedness plan [1]. Mitigating the potential impact of an influenza pandemic may be accomplished by non-pharmaceutical (e.g. closing schools, using facemasks, social distancing) and pharmaceutical interventions such as use of antiviral drugs and pandemic vaccines [2]. WHO coordinates global scientific research and development to ensure that antiviral drugs and pandemic vaccines are rapidly and widely available and that scientific understanding of the virus evolves quickly [3]. While pandemic vaccine production can only start once the pandemic virus has been recognized, pandemic vaccine capacity will be limited at least during the first phase of the pandemic [4]. The distribution of limited and progressively available vaccine supply will require strategies for vaccine prioritization, i.e. a concept defining which group(s) of people shall receive the pandemic vaccine first [5].

Considerations for vaccine prioritization will be different for each country, not only because of differences in vaccine availability and resources for administration of vaccine, but also because of differences in population structure and the organization of essential services [6]. However, differences in vaccine priority groups between neighbouring countries may lead to difficulties in implementation, especially when rationales underlying the concepts are not clearly communicated. Germany in particular has not less than 9 bordering countries. While the federal public health institute in Germany, the Robert Koch Institute (RKI) was approaching the neighbouring countries to learn about their strategies for vaccine prioritization, other countries also expressed their interest to have an overview of pandemic vaccine priorities internationally.

Recent reviews of pandemic preparedness assessments have been published that either briefly highlighted vaccine prioritization among several aspects of pandemic preparedness or provided aggregated data on vaccine prioritization in multiple developed and developing countries [7-9]. The goal of this review is to provide a thorough overview of pandemic vaccine prioritization concepts in the 27 European Union (EU) member states and the four non-EU countries of the Global Health Security Action Group (GHSAG).

## Methods

We explored the status of establishing pandemic influenza vaccine prioritization concepts in the 27 EU countries as of January 1^st ^2007 and the remaining non-EU countries belonging to the GHSAG (Canada, Japan, Mexico, United States). Between September and December 2006 we obtained information through the two following data sources: (i) for each country we first identified the national influenza pandemic plan available in the public domain. If the original or translated version was published in English, Danish, Dutch, French, German, or Swedish we extracted data directly. Otherwise we only obtained information through the second data source; (ii) for every country we contacted key persons involved in pandemic planning by either email and/or phone and/or fax.

We investigated the following questions:

(1) Does the country has a list of pandemic vaccine priority groups?

(2) Are pandemic vaccine priority groups ranked?

(3) What is the rationale for the pandemic vaccine prioritization concept?

(4) Were ethical committees involved in the establishment of the vaccine prioritization concept?

(5) Which types of institutions were involved in the development of vaccine priority groups?

Reported vaccine priority groups were first assigned to the following main categories that were similar to those in the guidelines from WHO [6]: 1) Health care workers (HCW); 2) Persons employed in positions that are essential for the maintenance of the infrastructure and public safety ("essential service providers"; ESP); 3) Individuals with high risk of death and severe complications ("high risk individuals"; HRI); 4) Healthy adults and children. We also categorized listed subgroups into these four main categories when applicable. Other groups that could not be assigned to one of these four main categories were listed in a separate table.

Similarly, the rationales for the vaccine prioritization concept chosen, were grouped into four categories which were again compatible with guidelines provided by the WHO [6]: 1) To reduce morbidity and mortality; 2) To maintain infrastructure, including functioning of the health care system; 3) To limit social disruption; 4) To limit economic losses. There was large variation in the phrasing and level of detailed specification of rationales. However, we attempted to put the reported rationales in the most appropriate category.

In December 2006 all identified key persons received a draft version of the tables included in this review and were able to comment on correct representation of their country and permission to publish the country specific data. Abbreviations can be found at the end of the manuscript.

## Results

### Vaccine priority groups

Except for Malta, the countries included in this review had a National Pandemic Influenza Plan published on the internet [see Additional file 1]. The language of the pandemic plans of all but seven countries (CY, FI, LV, MX, PT, RO, SI) were available in English, Danish, Dutch, French, German or Swedish and allowed direct extraction of the information.

Representatives from all 31 (100%) countries responded to our request for either updated or additional information on the information reported in the National Pandemic Influenza Plans available in public domain during the study period. Furthermore, representatives of 30 (98%) countries either confirmed the correctness or provided changes to the representation of their countries' data on the draft tables and the US referred to the information available in the public domain.

At least 19 (61%) countries (AT, BG, CA, CY, DE, DK, EE, FI, HU, IE, IT, JP, LT, MT, NL, PT, SE, UK, US) reported that ultimately the whole population shall be immunized with the pandemic vaccine. For countries that included "healthy adults and children" in their vaccine priority groups it was inferred that they are also planning to finally immunize the entire population. However, because vaccine will likely become progressively available while the pandemic has already started all but one (PT) of these 19 countries have already defined vaccine priority groups. A general statement frequently reported by the countries' representatives was that vaccine priority groups had been established to stimulate planning prior to a pandemic, but that these will be continually revised in light of new information that will be learnt once the pandemic virus is identified.

Except for Belgium, Latvia, Luxembourg, Mexico and Portugal twenty-six (84%) countries had established at least one vaccine priority group. Denmark and Greece did not list vaccine priority groups as such, but mentioned them as groups reflecting a discussion part in the National Pandemic Plan (DK) or as "suggested" (GR). We included information from these two countries in the description of vaccine priority groups in the 26 countries. Four of the five countries without defined vaccine priority groups may still do so as discussion about determining them was still ongoing (BE, PT), or may be established in the updated National Plan (LV) or when more information about vaccine production and availability is accessible (MX). (Table 1) Only Luxembourg will not determine any vaccine priority groups in advance of the pandemic. The number of vaccine priority groups as categorized by the countries varied from one to eight. This wide range is mainly due to differences in the level of detail in the definition of the specific vaccine priority groups. However, most of these vaccine priority groups could be categorized into HCW, ESP, HRI and "healthy adults and children". Among the 26 countries that defined vaccine priority groups 23 (88%) agreed to prioritize HCW, ESP and HRI (Table 1). There were three exceptions: Finland (HCW only), Germany (HCW and ESP) and the Netherlands (HCW and HRI).

**Table 1:** Establishment of Vaccine Priority Groups in EU^A ^and GSHAG^B ^countries

**Countries**	**Source(s) data abstraction included in this review**			**No. of main groups**	**Presence of list of subgroups within 3 main groups (Table 2)**	**Ranking of groups**	**Ethic committees/consultant involved**	**Intention to vaccinate whole population^C^**	**Pandemic vaccine priority groups^D^**				
	**Pandemic plan**	**Email fax**	**Phone**						**(1) Healthcare workers**	**(2) Essential service providers**	**(3) High risk individuals**	**(4) Healthy adults and children^E^**	**Other (Table 3) ^F^**
AT^A^	yes	yes	yes	3	no^1a^	no	yes^1b^	yes	yes	yes	yes	no	no
BE^A^	yes	yes	yes	discussion	not applicable	not applicable	no	unknown					
BG^A^	yes	yes	no	3	yes	yes	no^I^	yes^3^	yes	yes	yes	yes	no
CA^B,2^	yes	yes	no	5^H^	yes	yes	no^I^	yes	yes	yes	yes	yes	no
CY^A^	no^G^	yes	no	5^H^	unknown	yes	yes	yes	partly^4^	yes	yes	yes	no
CZ^A,^	yes	yes^5^	no	3	yes	unknown	unknown	unknown	yes	yes	yes	no	no
DK^A,6^	yes	yes	yes	7^H^	yes	yes, 2 groups^6^	no	yes	yes	yes	yes	yes^J^	no
EE^A^	yes	yes	no	3	yes	yes	no^I^	yes	yes	yes	yes	partly^K^	yes
FI^A,7^	no^G^	yes	no	1^7^	unknown	yes, 1 group^7^	yes	yes	yes^7^	no^7^	no^7^	no	no
FR^A,B,8^	yes	yes	yes	3	no	no	no	unknown	yes	yes	yes	no	no
DE^A,B^	yes	yes	yes	2^9a^	no^9b^	yes	no	yes	yes	yes	no	no	no
GR^A,10^	yes	yes	no	5^10^	unknown	discussion^10^	yes	unknown	yes^10^	yes^10^	yes^10^	no	yes^10^
HU^A^	yes	yes	no	3	no	yes	no	yes	yes	yes	yes	no	no
IE^A^	yes	yes	no	7^H^	yes	yes	no^I^	yes	yes	yes	yes	yes^J^	yes
IT^A,B^	yes	yes	no	6^H^	yes	yes	no	yes	yes	yes	yes	yes	yes
JP^B,11^	yes	yes	yes	6^H^	discussion	discussion	no	yes	yes	yes	yes	yes	yes
LV^A,12^	no^G^	yes	no	0^12^	not applicable	not applicable	unknown	unknown					
LT^A,13^	yes	yes	no	4	yes	discussion	no	yes	yes	yes	yes	no	no
LU^A^	yes	no	yes	0	not applicable	not applicable	unknown	unknown					
MT^A^	no^14a^	yes	no	8^H14b^	no	yes	no	yes	yes	yes	yes	yes	yes
MX^B,15^	no^G^	yes	no	0	not applicable	not applicable	yes	unknown					
PO^A,16^	yes	yes	no	2	yes	no	unknown	unknown	yes	yes	yes	partly^K^	no
PT^A^	no^G^	yes	no	discussion	unknown	discussion	yes	yes					
RO^A^	no^G^	yes	no	5	yes	yes	yes	unknown	yes	yes	yes	partly^K^	yes
SK^A^	yes	yes	no	4	yes	no	no	unknown	yes	yes	yes	no	yes
SI^A^	no^G^	yes	no	4	no	yes	no^I^	unknown	yes	yes	yes	partly^K^	no
ES^A^	yes	yes	no	4^H^	discussion	yes	no	discussion	yes	yes	yes	yes	no
SE^A^	yes	yes	no	7^H^	no	no	yes	yes	yes	yes	yes	yes	yes
NL^A^	yes	yes	yes	4^H^	yes	yes	no	yes	yes	no	yes	yes	no
UK^A,B,17^	yes	yes	no	7^H^	yes	yes	yes	yes	yes	yes	yes	yes^J^	yes
US^B,18^	yes	no	yes	4^H^	yes	yes	yes	yes	yes	yes	yes	yes	yes

**Summary**	**23/31 (74%)**	**29/31 (94%)**	**9/31 (29%)**	**26/31 (84%)**	**14/26 (54%)**	**17/26 (65%)**	**10/31 (32%)**	**19/31 (61%)**	**26/26 (100%)**	**24/26 (92%)**	**24/26 (92%)**	**13/26 (50%)**	**11/26 (42%)**

^A ^Member State of European Union (EU); ^B ^Member of Global Health Security Action Group (GSHAG); ^C ^'yes'indicates that either National Pandemic Plan specifically reports the aim to vaccinate whole population and/or National Pandemic Plan includes 'healthy adults and children' as one of the vaccine priority group(s) and/or country representatives specifically reported this information; 'discussion' indicates country representative specifically reported that vaccination policy is still under discussion, 'unknown' indicates that this item is not specifically reported; ^D ^The issue of prioritisation arises only in the situation of vaccine shortage or when vaccines become available over time; ^E ^'yes refers to those countries either specifying 'healthy adults and children' as vaccine priority group(s) and those countries reporting 'offer to all' as vaccine priority group; ^F ^Vaccine priority groups that could not be categorized in the four most common vaccine priority groups as described in this table; ^G ^National Pandemic Plan available in public domain in language other than English, Danish, Dutch, French, German or Swedish; ^H ^Including a category referring to vaccination of whole population; ^I ^Ethics considerations will be involved in the future; ^J ^Included under the vaccine priority group 'offer to all'; ^K ^Countries have included 'children' under 'high risk individuals'

^1a ^(AT) National Pandemic Plan serves as a framework to guide each of the nine states to develop specific plan including vaccine priority subgroups; ^1b ^No specific ethical committee involved but ethical considerations have been involved;

^2 ^(CA) Advancements in knowledge and technology are currently being considered by the Pandemic Vaccine Working group and may result in the revision of vaccine priority groups in updated Pandemic Plan in 2007

^3 ^(BG) Specified in draft national influenza preparedness plan of Bulgaria as "aim is to cover gradually the maximum part of the population";

^4 ^(CY) Specified as people working in the Health Sector involved with the distribution of vaccines and antivirals;

^5 ^(CZ) Information received by email is from the unpublished National Pandemic Plan of the Czech Republic 2006;

^6 ^(DK) Prioritisation of vaccine strategies will be decided during the course of a pandemic, the information in the table reflects the discussion paper in the pandemic plan;

^7 ^(FI) Irrespective of the virus one vaccine priority group is determined in advance;

^8 ^(FR) Working groups are updating strategies using pre-pandemic modelling but also accurate survey of the population and age-groups most affected by virus, associated with models of progressive availability of the vaccine in relation with the kinetics of a pandemic will determine the vaccine priority groups;

^9a ^(DE) Two main vaccine priority groups have been determined, discussion related to criteria of establishing further vaccine priority groups are under discussion ^9b ^National Plan serves as a framework to guide each of the 16 states to develop specific plan including vaccine priority subgroups.

^10 ^(GR) Is in the process of discussing and further refining vaccine priority groups, the information in the table includes "suggested priority groups" as published in National Pandemic Plan;

^11 ^(JP) Prioritisation is under discussion and the table includes information in draft guidelines available for public discussion and comments;

^12 ^(LV) Influenza Pandemic Preparedness Plan of Latvia will be updated in 2007;

^13 ^(LT) Vaccine priority groups will be revised in the "near future" [as of October 26^th ^2006];

^14a ^(MT) National Influenza Pandemic Standing Committee has decided on vaccine priority groups and currently the plan prepared by this committee is being approved and amended by the Director General; ^14b ^Seven of eight reported vaccine priority groups have been categorized under four main categories included in this table;

^15 ^(MX) Current National Preparedness and Response Plans does not include a scenario in which pandemic vaccine is available; this aspect may be updated as information on vaccine production and availability becomes available.

^16 ^(PL) More specific and detailed strategy under discussion;

^17 ^(UK) Plan is currently under revision, including more modelling of immunisation strategies;

^18 ^(US) Revision of order prioritisation of tiers is expected in spring 2007.

Thus, **HCW **were included as vaccine priority group in all these 26 countries. Cyprus specified "individuals involved with the distribution of vaccines and antivirals" as one vaccine priority group and "people working in essential services" as another vaccine priority group. This latter group may by definition include HCW but specific information about subgroups was not available (Table 1). The most common subgroups specifically listed under HCW included paramedics, ambulance and emergency services as well as in-patient health care workers (Table 2).

**Table 2:** Common reported subgroups within three main vaccine priority groups as listed in National Influenza Pandemic Plans ^A^

	**BG**	**CA**	**CZ**	**DK**	**EE**	**IE**	**IT**	**LT**	**PL**	**RO**	**SK**	**NL**	**UK**	**US**	
**Health Care Workers in: ^B^**	.	.	.	.	.	.	.	X	.	.	.		.	X^F^	
Clinical laboratories, pharmacies	X	X	X	.	X	.	.	.	.	X	.	X	.	.	6 (43%)
Paramedics, emergency services	X	X	X	X	.	X	X	.	X	X	.	X	X	.	10 (71%)
Residential care homes for elderly	.	X	.	.	.	X	.	.	.	.	.	.	X	.	3 (21%)
Social institutions (e.g. day care, "elderly houses")	X	.	X	X	.	.	.	.	.	.	X	.	.	.	4 (29%)
Public health agencies/authorities	X	X	X	.	X	.	X	.	.	X	.	.	.	.	6 (43%)
Long term health care facilities	.	X	X	.	.	.	X	.	X	.	X	X	.	.	6 (43%)
Out-patient health care facilities	X	X	X	X	.	.	X	.	.	X	.	X	.	.	7 (50%)
In-patient health care facilities	X	X	X	X	.	X	X	.	X	X	.	X	X	.	10 (71%)
															
**Essential service providers ^B^**															
															
*Key persons in government, emergency response, defence and security*	X	X	X^C^	X^D^	.	X^E^	.	.	X	:	X	.	X^E^	X	
Police	X	X	X	.	X	X	X	.	X	X	X	.	X	.	10 (71%)
Armed forces	X	X	X	.	X	X	X	.	X	X	X	.	X	.	10 (71%)
Fire fighters	X	X	X	.	.	X	X	.	X	X	X	.	X	.	10 (71%)
Government	X	X	X	.	.	.	.	.	.	X	X	.	.	X	6 (43%)
Emergency response decision makers	X	X	.	.	X	.	X	X	.	.	.	.	.	X	6 (43%)
Border guards and custom officers	X	.	.	.	X	.	X	.	X	X	.	.	.	.	5 (36%)
Defined national authorities	X				X					.					2 (14%)
															
*Public Sector*		X^C^	.	.	.	.	X	X^E^	.		.	.	.	X^D^	
Utility workers (e.g. power, water, sewage system)	X	X	X	.	X	X	X	X	.	X	X	.	X	X	11 (79%)
Funeral services/mortuary personnel	X	X	.	.	.	X	.	.	.	X	.	.	X	X	6 (43%)
Transport (e.g. fuel, water, food, medical supplies)	X	X	X	.	.	.	X	.	X	X	X	.		X	8 (57%)
															
**High risk individuals ^B^**															
Groups identified in pandemic as being at increased risk for complications	X	X	.	X	.	X	.	.	.	.	.	X	X	.	6 (43%)
Sub-groups similar to those where vaccination is recommended during seasonal influenza	X	X	X	.	X	X	X	X	X	X	X	X	X	X	13 (93%)
Pregnant women	.	X	.	.	X	.	.	.	X	.	.	.	.	X	4 (29%)
Pregnant women in the third trimester	.	.	.	.	.	X	.	X	.	.	.	X	X	.	4 (29%)
Children 6 to 23 months	.	X	.	.	.	X	X	.	.	X	.	.	.	X	4 (29%)
Children and youth 6 months to 18 years	.	.	.	.	X	.	.	.	X	.	.	.	.	.	2 (14%)

^A ^Table includes countries which listed subgroups in their published National Pandemic Influenza Plans that either met the language criteria (see Methods) or provided detailed information on request; ^B ^Due to countries variation in the terminology among countries overlaps between subgroups may exist. Table includes subgroups if categorized by countries as belonging to one of the main groups; ^C ^National Pandemic Influenza Plan reports a comprehensive list of subgroups which is not completely summarized in this table; ^D ^National Pandemic Influenza Plan reports a comprehensive list of groups which could be considered and is not summarized in this table. "Decisions on priority groups will be made when the characteristics of the new pandemic virus is known and according to ongoing work on vulnerability in specific key sectors"; ^E ^Unmarked subgroups are not necessarily excluded because the National Pandemic Influenza Plan only reports a few examples; ^F ^No further specification.

Except for Finland and the Netherlands, **ESP **were included as vaccine priority group by 24 (92%) of 26 countries that had established at least one vaccine priority group. Identified subgroups belonging to this category were e.g. key persons in government, emergency response, defence and security as well as in the public sector (for example utility workers, Table 2).

Only Finland and Germany did not include **HRI **as vaccine priority group, thus HRI were included by 24 (92%) of the 26 countries. In many countries the definition of this group is very similar to the one used for seasonal influenza recommendations, but not all countries specifically listed all subgroups in this context. Of note, six countries (BG, CA, DK, IE, NL, UK) report specifically that the exact types of risk group will need to be identified during the pandemic (through investigations, studies or surveillance).

**Healthy adults and children **were included as one or two vaccine priority groups by 13 (50%) countries (BG, CA, CY, DK, ES, IE, IT, JP, MT, NL, SE, UK, US), including 3 countries (DK, IE, UK) that intend to "offer vaccination to all individuals not belonging to any other vaccine priority group". Estonia, Poland, Romania and Slovenia reported to vaccinate children (and not healthy adults) as they considered them to belong to HRI.

**Other vaccine priority groups **have been established in 11 (42%) of 26 countries (EE, GR, IE, IT, JP, MT, RO, SE, SK, UK, US). However, partially they represent just an extension of the above named first three main groups (HCW, ESP, HRI) for example long term care facility residents (RO) may overlap with HRI and selected industries may overlap with ESP (IE, UK). Other groups defined were e.g. staff of workplaces where many people gather (SK), household contacts of immunocomprised persons (SK, US), pandemic vaccine manufacturers (RO, US) and a 'wild card' selected age groups based on National or WHO recommendations as reported by three countries (IE, MT, UK) (Table 3).

**Table 3:** List of vaccine priority groups other than health care workers, essential service providers, high risk individuals and healthy adults/children

	**EE**	**GR**	**IE**	**IT**	**JP**	**MT**	**RO**	**SK**	**SE**	**UK**	**US**	**N = 11 (100%)**
*Reported by countries as specific vaccine priority group*												
Long term care facility residents^A^							X					1 (9%)
Individuals living in "closed communities" (*e.g. nursing homes*)^A^	.	X^1^	.	.	.	.			.	.	.	1 (9%)
Individuals in crowding area (*e.g. schools*)	.	X^1^	.	.	.	.	.	.	.	.	.	1 (9%)
Staff of workplaces where many people gather (*e.g. banks, shops*)	.	.	.	.	.	.	.	X	.	.	.	1 (9%)
"Selected age groups, depending on national and/or WHO advice"^B^	.	.	X	.		X	.	.	.	X	.	3 (27%)
Selected industries (e.g. pharmaceuticals)^C^	.	.	X	.		.	.	.	.	X	.	2 (18%)
												
*(Sub) groups not clearly categorized as belonging to main groups*												
Pandemic vaccine manufacturers^D^	.	.	.	X^2^	.	.	X^4^	.	.	.	X^5^	3 (27%)
Household contacts of immunocompromised persons^E^	.	.	.	.	.	.	.	X	.	.	X	2 (18%)
Household contacts of high-risk patients	X	.	.	.	.	.	.	.	X	.	.	2 (18%)
Household contacts of children	.	.	.	.	.	.	.	.	.	.	X	1 (9%)
Seniors					X^3^							1 (9%)

^A ^Specified as additional vaccine priority group to the "high risk individuals"; while in other countries these individuals may be included in seasonal vaccination group; ^B ^Vaccination policy might be revised in the event of one or more age groups being more severely affected in a pandemic; ^C ^Reported as separate vaccine priority group; while in other countries these individuals might have been included, but had not been listed, under the "essential workers"; ^D ^Countries have specifically reported this (sub) group while in other countries these might have been included, but had not been listed, under "essential workers"; ^E ^This category also includes "family members of persons with impaired immunity" (SK); ^1 ^Currently in the process of discussing and refining further priority groups, information in the table includes "suggested" priority groups as published in the National Pandemic Plan; ^2 ^Not listed in National Pandemic Plan, but updated information received from country representative; ^3 ^Reported as separate vaccine priority groups in draft guidelines; ^4 ^Specified as separate (1 of 6) vaccine priority group; ^5 ^Specified as 'subtier' belonging to one of four 'tiers' and includes also antiviral manufacturers

### Priority ranking

Ranking of at least one vaccine priority group was done by 17 (65%) of 26 countries (BG, CA, CY, DE, DK, EE, IE, FI, HU, IT, MT, NL, RO, SI, ES, UK, US) which had established at least one vaccine priority group.

Fifteen (88%) (BG, CA, DE, DK, EE, ES, FI, HU, IE, IT, MT, RO, SI, UK, US) of these 17 countries decided that HCW with close contact to influenza patients should be vaccinated first; in most countries (BG, CA, EE, ES, DE, HU, IE, IT, MT, UK) followed and/or ranked equally by ESP and subsequently HRI (BG, CA, EE, ES, IE, IT, UK) (Table 4). Except for the very young children which might receive vaccination because they belong to the seasonal influenza vaccination group, children and healthy adults are not ranked at the top in any of the countries. However, in Japan, a discussion on the ranking of children is ongoing and will depend on the chosen rationale being either to prevent severe cases from dying or "to save people considering future life" (email communication). At the time of writing, ranking was either under discussion (GR, LT, JP); information unavailable (CZ); or not done (AT, FR, PL, SE, SK) by 9 (35%) of 26 countries that had established vaccine priority group(s). These, and also other, countries reported that establishment and ranking of vaccine priority groups is constantly under discussion and final decision will depend on the specific epidemiological characteristics of the new pandemic virus and the pandemic vaccine availability.

**Table 4:** Ranking of vaccine priority groups ^A^

Countries	**1^st ^priority**	**2^nd ^priority**	**3^rd ^priority**	**4^th ^priority**	**≥5^th ^priorities**
BG^B^	Health care workers Essential service providers	High risk individuals	Healthy adults and children		
CA	Health care workers	Essential service providers	High risk individuals	Healthy adults	Children
CY	Health care workers involved with vaccines and antiviral distribution	Essential service providers	High risk individuals	Healthy adults^C^	Individuals aged 2–18 yrs^C^
DK^D^	High risk individuals Health care workers		-	-	-
EE^E^	Health care workers	Essential service providers	High risk individuals	-	-
FI^F^	Health care workers	-	-	-	-
DE	Health care workers	Essential service providers	-	-	-
HU	Health care workers	Essential service providers			
IE	Health care workers	Essential service providers	High risk individuals	Individuals aged > 65 yrs.	5) Selected industries 6) Selected age groups, 7) Offer to all
IT	Health care workers	Essential service providers	High risk individuals	Healthy individuals aged 2–18 yrs.	Healthy adults
MT	Health care workers Essential service providers	Specific age groups with high complication risk	Individuals aged > 65 yrs with chronic diseases^G^	Individuals aged 2–64 yrs. with chronic diseases^G^	5)Individuals > 65 years without chronic disease 6) infants aged 6–24 months^G^ 7) Healthy individuals aged 2–16 yrs.^G^ 8) Healthy adults aged 17–64 yrs.^G^
RO	Health care workers	Vaccine manufacturers and distribution staff	Essential service providers	Long term care facilities residents	High risk individuals
SI	Health care workers	Workers involved in pandemic control	High risk individuals	-	-
ES	Health care workers	Essential service providers	High risk individuals	Healthy adults and children	
NL^H^	"Highest risk" individuals	Health care workers	Specific Pandemic Risk groups	Healthy adults and children	-
UK	Health care workers	Essential service providers	High risk individuals	Individuals aged > 65 yrs.	5) Selected industries 6) Selected age groups, 7) Offer to all
US^I^	Vaccine and antiviral manufacturers Health care workers (Tier 1a)	High risk individuals (Tier 1b)	Pregnant woman, household contacts of severely immunocompromised persons, household contacts of children (Tier 1c)	Public health emergency response, Key governmental leaders (Tier 1d)	5) Healthy individuals aged ≥ 65; individuals aged 6 months to 64 yrs. with 1 high-risk condition; healthy infants aged 6–23 months (Tier 2a) 6) Critical infrastructure group (Tier 2b) 7) Offer to all

^A ^The issue of prioritisation arises only in the situation of absolute or temporal vaccine shortage (or need for time wise stratification due to production time-frame); ^B ^A final decision regarding priorities will be taken under the conditions of the pandemic; ^C ^In view of the large numbers of persons among healthy adults and children a decision to vaccinate them will depend on the availability of vaccines, in view of the big numbers of persons covered by these groups; ^D ^Includes only the two vaccine priority groups who are determined as for prioritisation in advance: decisions on further prioritisation by one of five vaccination strategies as reported in the National Pandemic Influenza Preparedness Plan will be made during the course of the pandemic; ^E ^Estonian mass vaccination policy foresees four immunization strategies of which implementation depends on vaccine availability; the table includes the prioritization y in two of four strategies assuming limited vaccine; ^F ^Includes the one vaccine priority group determined in advance; further decisions will be made when more information about the virus is available; ^G ^In the case that all age groups are affected similarly then these groups will be prioritised (as decided by Malta's National Influenza Pandemic Standing committee and currently being approved and amended by the Director General); ^H ^The Health Council Committee defined four groups of people that, on medical grounds are most in need of vaccination. "Group 1" will be vaccinated first; in this table this group is depicted as 'highest complication individuals'; the category "Specific Pandemic Risk Groups" probably shows overlap with the "Group 2–4" as defined by the Health Council Committee; healthy adults and children will be vaccinated when enough vaccine is available ^10^; ^I ^Vaccination priority groups are categorised into 4 tiers including subtiers which will be vaccinated with increasing availability of vaccine. Description in this table is limited to tear 1–2. Currently discussion is ongoing to update the ranking of vaccine priority groups.

### Rationales

Except for the Czech Republic, Japan, Poland and the Slovak Republic rationales for prioritization were provided by 22 (85%) of 26 countries that established vaccine priority groups (Table 5). There was large variation in the phrasing and level of detailed specification of rationales. However, we attempted to put the reported rationales in the most appropriate category.

**Table 5:** Overview of rationales considered in EU and GSHAG countries to define vaccine priority groups ^A^

	AT	BG	CA	CY	CZ	DK	EE	FI	FR	DE	GR	HU	IE	IT	JP	LT	MT	PL	RO	SK	SI	ES	SE	NL	UK	US
**To reduce morbidity and mortality (1)**	X	X^B^	X	.	.	X^E^	.	.	.	X	.	.	X	.	.	.	.	.	X	.	X	X	.	.	X	.
To save years of life	.	.	.	.	.	.	.	X^F^	.		.	.	.	.	.	.	.	.	.	.	.	.	.	.	.	.
To prevent illness in the general population	X	.	X	.	.	X^E^	.	X	.		.	.	.	.	.	.	.	.	.	.	.	.	.	X	X	X
																										
*directly*																										
- in individuals most vulnerable to severe illness	.	X	X	.	.	X^E^	.	.	X		.	.	.	.	.	X^I^	X	.	.	.	.	.	X	X	X	X
- in age groups most vulnerable to severe illness	.	.	.	.	.	.	.	.	.		.	.	.	.	.	.	X	.	.	.	.	.	X	.	.	.
- in health care workers at increased exposure risk*	.	.	X	.	.	X^E^	X	X	X	X	X	X	.	x	.	.	.	.	X	.	.	.	X	X	X	X
																										
*indirectly*																										
- by preventing or minimising the spread of infection		.																						X		
*to *general population	.	.	X	.	.	.	.	.	.		.	.	.	.	.	.	.	.	.	.	X	.	.	.	X	
*to *high risk individuals	.	.	.	.	.	X^E^	.	.	.	X	.	.	.	.	.	.	.	.	.	.	.	.	X	.	X	
*to *immunocompromised and children	.	.	.	.	.	.	.	.	.		.	.	.	.	.	.	.	.	.	.	.	.	.	.	.	X
																										
**To maintain infrastructure and health care system (2)**	X	X	.	.	.	.	.	.	.	X	.	.	.	X^G^	.	.	.	.	.	.	.	X	.	.	X	X
By maintaining the major work force	.		X	.	.	.	.	.	.		.	.	.	.	.	.	.	.	.	.	.	.	.	.	.	.
By maintaining the essential community services	.	X	X	X	.	X^E^	X	.	X		X	X	X	X	.	X	X	.	X	.	X	.	X	X	X	X
*which may include the following specified rationales:*																										.
*maintain *service to implement pandemic response	.	.	.	X^C^	.	.	X	.	X.		.	.	.	.	.	.	.	.	.	.	X	.	.	.	.	X
*maintain *vaccine program	.	.	X	.	.	.	.	.	.		.	.	.	.	.	.	.	.	X	.	.	.	X	.	.	X
*maintain *essential health service response*	.	.	X	.	.	X^E^	X	X	X		X	X	.	X^G^	.	.	X	.	X	.	.	.	X	X	X	X
*maintain *security	.	.	.	.	.	.	.	.	.		.	.	.	.	.	X	.	.	.	.	.	.	.	.	.	
																										
**Limiting social disruption (3)**	.	X	.	.	.	.	.	.	.	.	.	.	.	.	.	.	.	.	.	.	.	.	.	.	.	.
**Limiting economic losses (4)**	.	X	.	.	.	.	.	.	.	.	.	.	.	.	.	.	.	.	.	.	.	.	.	.	.	.
																										
*Any rationale reported?*	X	X	X	X	^D^	X^E^	X	X	X	X	X	X	X	X	^H^	X	X	^H^	X	^J^	X	X	X	X	X	X
*Any rationale to 1?*	X	X	X	.	.	X^E^	X	X	X	X	X	X	X	X	.	X	X	.	X	.	X	X	X	X	X	X
*Any rationale to 2?*	X	X	X	X	.	X^E^	X	X	X	X	X	X	X	X	.	X	X	.	X	.	X	X	X	X	X	X
*Any rationale to 3?*	.	X	.	.	.	.	.	.	.	.	.	.	.	.	.	.	.	.	.	.	.	.	.	.	.	.
*Any rationale to 4?*	.	X	.	.	.	.	.	.	.	.	.	.	.	.	.	.	.	.	.	.	.	.	.	.	.	.

* To provide a complete overview and deal with different terminology of countries in specifying this rationale we have marked the rationale in both categories for each country specifying this rationale;

^A ^Level of detailed specification of rationales varied between the countries, as a result rationales in this table may overlap; ^B ^In more detail specified as "to reduce complications, necessity of hospitalizations and lethality"; ^C ^Specified followed: 'Covering those who are going to be actively involved in mastering a possible pandemic'; ^D ^National Influenza Pandemic Plan includes the following general statement: 'Vaccine Priority groups have been established according to clear, medical, social and economic reasons'; ^E ^National Influenza Pandemic Plan describes 5 strategies which are the basis for the different rationales to decide which vaccine groups will be prioritized; during the course of the pandemic decision will be made which strategies to follow; ^F ^Number of potential life years saved will be the most important determining factor for prioritization; ^G ^National Influenza Plan does not contain specific rationales, but rationales which set priorities as represented in this table were given through personal communication; ^H ^Rationales are under discussion; ^I ^Specified as individuals at highest risk of influenza; ^J ^National Influenza Pandemic plan does not contain specification of rationales for priority setting; personal communication reveals that the main principle is "to involve groups in the highest risk of infection, complication and death and groups in the population that are important for maintaining the public life".

The category **"To reduce morbidity and mortality" **included rationales reported by 21 (95%) of 22 countries that reported any rationale, only Cyprus did not report this rationale. The general non-specific rationale "to reduce morbidity and mortality" was reported by 10 (45%) of 22 countries (AT, BG, CA, DK, DE, ES, IE, RO, SI, UK); and the rationale "to prevent illness in the general population" was also reported by 4 (AT, CA, DK, UK) of these 10 countries and by 3 other countries (FI, NL, US) not specifically reporting the general rationale. We included the rationale "to save years of life" as reported by Finland also under this general category. We further dichotomized this category into "directly" and "indirectly" as morbidity and mortality can be reduced through vaccinating the respective group of persons (direct protection) or by vaccinating those who could transmit the virus to them (indirect protection). For example 10 countries (BG, CA, DK, FR, LT, MT, NL, SE, UK, US) gave direct protection as (one of) the underlying rationale(s) for their prioritization of persons considered to be most vulnerable to severe illness. Three countries (CA, SI, UK) intend to prevent transmission to the general population and minimal four countries (DK, DE; SI, UK) to high risk individuals by vaccinating those individuals most likely to transmit the virus to them.

The category **"To maintain infrastructure and health care system" **includes rationales reported by all 22 countries that provided rationales (AT, BG, CA, CY, DE, DK, EE, ES, FI, FR, GR, HU, IE, IT, LT, MT, NL, RO, SI, SE, UK, US). These were divided into two broad categories: maintenance of the work force and maintenance of essential community services. The latter was specified by overall 19 countries and compromised for example maintenance of the essential health service response (14 countries: CA, DK, EE, FI, FR, GR, HU, IT, MT, NL, RO, SE, UK, US) and maintenance of the infrastructure to implement the pandemic response (5 countries: CY, EE, FR, SI, US). Maintenance of the work force was named explicitly only by Canada.

Only seven countries (BG, CA, DE, DK, IE, UK, US) clearly associated each vaccine priority group with the specific rationale underlying the inclusion of the specific group. At least five countries (CY, EE, HU, RO, IT) did not specify rationales for all established vaccine priority groups. The inclusion of a specific vaccine priority group may be rationalized different by countries. E.g. in Estonia and Poland children are included under the rationale to protect high risk individuals and at least three countries (CA, NL, US,) specifically reported that children are only vaccinated when sufficient vaccine is available to vaccinate the entire population anyway. Canada intends to vaccinate children specifically to limit spread of infection.

### Decision making process

Coordination of the pandemic preparedness is in most countries under the responsibility of the Ministry of Health. Frequently working groups of experts or advisory committees for vaccination advised the government in the development of the pandemic preparedness plans. Six countries (19%) (DE, FR, IE, NL, UK, US) have included references to pre-pandemic modelling in their pandemic plan and/or referred to ongoing (or, when the pandemic has begun: real time) mathematical modelling that might result in future changes of vaccine priority groups. Ten (32%) of 31 countries have consulted and involved ethical experts (AT, CY, FI, GR, MX, PT, RO, SE, UK, US) to guide decisions related to vaccine prioritization, five further countries (16%) (BG, CA, EE, IE, SI) reported the intent to include ethical aspects in future discussions.

## Discussion

The objective of this review was to provide an overview of pandemic vaccine priority groups in 27 EU member states and the 4 non-EU countries that are members of the GSHAG. We included these countries as they are part of organized structures (e.g. Health Security Committee, European Union) with the goal to strengthen public health preparedness and response globally to not only threats of biological, chemical, radio-nuclear terrorism but also pandemic influenza [11,12]. A multinational comparison between countries may be useful to e.g. identify large differences in prioritization between cross bordering countries which may result in either harmonisation or proactively explaining the potential differences. For example we found a large difference in ranking priority between the neighbouring countries Germany and the Netherlands. The Netherlands is the only country that has not top ranked health care workers but the "highest complication individuals"; this latter group is in Germany's National Pandemic Plan, but not included as one of the two established vaccine priority groups. However, we do not aim to provide the most suitable prioritization concept. We rather wish to provide an overview of approaches in the wider European perspective and want to discuss some general aspects in more detail. This might help countries to put themselves into the international context and/or to assist them should they be in the process to formulate or overhaul pandemic vaccine prioritizations concepts.

Nineteen (61%) of the countries state that they intend to finally vaccinate the whole population, but most admit that progressive vaccine availability will make some form of prioritization unavoidable. Twenty six (84%) of the 31 surveyed countries had defined vaccine priority groups which were fairly similar. Commonly defined vaccine priority groups included HCW, ESP and HRI and are thus consistent with WHO guidelines [6]. Approximately half of these countries have defined subgroups among those three main groups, some, e.g. Canada, Czech Republic and Denmark specified many groups in great detail. This may reflect their intention to respond to two of the major challenges in pandemic vaccine prioritization: logistical administration and acceptability. It seems likely that the better priority groups are defined in advance of the pandemic the easier it is to identify eligible individuals or conversely, ask those who are not or not yet eligible to hold out to the point of time when it is their turn. Identifying HCW and ESP might be possible through personal registers. However, identification of HRI might be more complex. While in e.g. the Netherlands those individuals belonging to the high risk influenza complication group are yearly invited by their general practitioner to receive seasonal influenza vaccination, such a register has not been established in Germany. Owing to difficulties in prioritization on the basis of chronic diseases, age is often a surrogate for identifying those at greatest risk of complications [6]. Therefore, a pragmatic solution might include simple criteria by e.g. grouping HRI by year of birth. In addition, early communication and transparency of prioritization may aid greatly in the acceptability of concepts within the population. We did not observe a clear association between population size and the number of established vaccine priority groups. Austria and Germany, the only two EU countries with a federal government, state that the National Pandemic Plan serves as a framework to guide the pandemic preparedness in each of the individual states. Both federal EU-countries leave the specification of subgroups to the responsibility of the individual states. This is in contrast to two of the three non-EU GSHAG countries with a federal structure included in this review. Both Canada and the USA have included extensive lists of subgroups to guide the state health departments. Thirteen (93%) of 14 countries that foresee prioritization of HRI and have reported subgroups, have defined them similarly or identically to those for whom seasonal influenza vaccination is recommended, but 6 (43%) also want to keep their flexibility to give groups priority who will be identified in the pandemic as being at increased risk for complications. In this context children or subgroups of children are only covered within the HRI group. Two-thirds (65%) of the countries having established vaccine priority groups ranked at least one of these groups, but also kept the flexibility to change ranking when more information about risk groups and vaccine availability will be accessible

Broad agreement exists among countries regarding ranking of the vaccine priority groups with HCW as the first priority, ESP as second and HRI as third, in line with WHO recommendations [6]. None of the 14 countries that included children as priority (sub)group, and ranked priority groups, prioritized the vaccination of children above the previously mentioned three groups unless they are regarded as HRI.

Apparent discrepancies exist between WHO recommendations as well as the low priority given to vaccination of children on one hand with published results from mathematical modelling on the other hand that rather favour prioritization of children [13-15]. Ferguson *et al. *modelled the effect of a staged vaccination programme at the beginning of a pandemic in the USA and showed a maximum effect on the reduction of transmission if children are vaccinated first [13]. Similarly, Germann *et al. *concluded that schoolchildren should receive high priority for vaccination, unless other strategies, such as closing schools, are implemented to limit the contact rates among children [14]. Longini *et al*. estimated that vaccinating 80% of the children aged less than 19 years will be almost as effective as vaccinating 80% of the population, and this strategy would be the most efficient use of the vaccine [15] The 2004 publication from WHO and likely also the working process of most pandemic plans reviewed here preceded these publications [6]. WHO stated that there is an absence of evidence that use of inactivated vaccine in children will reduce the spread of a pandemic in the community. It is unclear however, if recommendations were drastically different since a substantial reduction of transmission among and from children might be achieved through social distancing measures of children, such as closure of schools [14]. While 6 countries (19%) have included references to pre-pandemic modelling and/or referred to ongoing mathematical modelling, we cannot conclude that pre-pandemic modelling has not played a role in the decision making in other countries because we did not systematically ask this specific question.

Rationales for prioritization were provided by 85% of the countries that had established vaccine priority groups. Yet again, clearly stating the objectives and rationales of vaccine prioritisation will be important for acceptance in the general population. Of note, prioritization of HCW may be justified by three rationales: (1) to protect them directly because they are at increased risk (reducing morbidity and mortality), (2) to prevent or minimise transmission to HRI (indirect reduction of morbidity and mortality), and (3) by maintaining infrastructure and health care system.

Ideally the goals of prioritization need to be stated clearly and unambiguously. Meltzer *et al *showed that differences in prioritization goals or rationales can result in completely different prioritization ranking [16]. E.g. if preventing the greatest number of deaths is the most important goal, adults in the working age (20–64 years old) with the highest risk for severe illness should be vaccinated first. However, if maximizing economic returns is the highest priority HRI aged 0 to 64 years should be vaccinated first. Also the importance of the specificity of goals was shown by Meltzer. For example, the commonly formulated goal to 'prevent deaths', is not precise enough because different priority lists can be drawn up by using death rates (elderly HRI) versus the total number of deaths (working age HRI) [16]. Thus, criteria for prioritization should be clear and defensible. Several countries have only partly (or not at all) specified the rationales for prioritization. In addition, it was not always obvious to make the link between rationales and priority groups, or differently stated which rationales were underlying the prioritisation of which specific group. For example, Bulgaria specified the rationale "to limit economic losses" but it was not clear which priority group was prioritized because of this.

Apart from epidemiological deliberations the allocation of scarce resources also requires ethical considerations. The importance of including ethics in pandemic preparedness was addressed during a recent WHO workshop [17]. Only 32% of the countries included in this review reported to have involved an ethic committee or consultant, and 16% reported the intention to include ethical aspects in future discussions. While for 13% of the countries information is unavailable the remaining 30% has not reported to have involved ethical consultants, it is unknown if these countries consider including ethics in the future.

A final note concerns the dynamic process of developing vaccine prioritization strategies as this might be influenced by e.g. availability of antivirals and developments in vaccine technology (e.g. the stockpiling of pre-pandemic H5N1 vaccines). These developments may change the perception of policy makers with respect to the need of defining or updating prioritization strategies.

## Conclusion

In the majority of the countries the establishment of vaccine priority groups, ranking and underlying rationales are in line with WHO recommendations. The first challenge in the prioritization of pandemic vaccines is public acceptability, which can be first addressed by involving ethical experts or an ethics committee in the decision making process. In this regard the result may even be less important than the process. In most public plans the criteria by which prioritized groups are identified are not easily recognizable. Goals of prioritization need to be stated clearly and criteria need to be linked with the groups that are to be prioritized. The resulting concept should then be communicated as clearly and precisely as possible including a detailed and unambiguous set of defined groups that will be prioritized and in which order. This approach should also aid in the management of the second major challenge: how prioritized individuals can be identified and how the vaccine can be administered to them. Registers may help to easily and rapidly identify and keep track of HCW and ESP. HRI could be simply grouped by year of birth, although there may be some HRI that are not captured adequately by this procedure.

Results of modelling exercises may and perhaps should play an increasing role in vaccine prioritization. Lastly, there is a need to build in flexibility and to be able to change policy intermittently (in the prepandemic phase) or perhaps even rapidly (in the pandemic) should information arise that supports modification of prioritization strategies. But for this purpose the rationales for prioritization ought to be clearly defined.

## Abbreviations

AT, Austria; BE, Belgium; BG, Bulgaria; CA, Canada; CZ, Czech Republic; CY, Cyprus; DE, Germany; DK, Denmark; EE, Estonia; ES, Spain; ESP, Essential Service Providers; EU, European Union; FI, Finland; FR, France; GHSAG, Global Health Security Action Group; GR, Greece; HCW, Health Care Workers; HRI, High Risk Individuals; HU, Hungary; IE, Ireland; IT, Italy; JP, Japan; LT, Lithuania; LV, Latvia; LU, Luxembourg; MT, Malta; MX, Mexico; NL, the Netherlands; PL, Poland; PT, Portugal; RO, Romania; SE, Sweden; SI, Slovenia; SK, Slovak Republic; UK, United Kingdom; US, United States.

## Competing interests

The author(s) declare that they have no competing interests.

## Authors' contributions

MS, UB, GK participated in the design of the study. MS collected the data. MS served as lead author of this report, UB contributed substantially to drafting and redrafting the report. WH, SR and GK contributed to redrafting the final version of the manuscript and have approved the final manuscript. All authors read and approved the final manuscript.

## Pre-publication history

The pre-publication history for this paper can be accessed here:



## Supplementary Material

Additional file 1 Pandemic influenza plans and relevant appendixes. This file provides references to the pandemic influenza plans and relevant appendixes and/or governmental acts of those countries included in the review. Click here for file
