# Supplementary material for: Prioritization strategies for pandemic influenza vaccine in 27 countries of the European Union and the Global Health Security Action Group: a review
Source: BMC Public Health. 2007 Sep 7;7:236. doi: 10.1186/1471-2458-7-236 (PMC2048949; doi:10.1186/1471-2458-7-236)
Supplement: Additional file 1 — Pandemic influenza plans and relevant appendixes. This file provides references to the pandemic influenza plans and relevant appendixes and/or governmental acts of those countries included in the review. [file 1471-2458-7-236-S1.doc]

**Pandemic influenza plans and relevant appendixes, (Date last accessed August 1^st^ 2007)**

**Austria** (November 2006) Influenza Pandemieplan Strategie für Österreich;

<http://www.bmgf.gv.at/cms/site/attachments/3/6/8/CH0019/CMS1126084167391/pp_inetversion12_06.pdf>

**Belgium** (July 2006) Belgium Preparedness Plan Influenza Pandemic; part 2 operational plan;

<http://www.influenza.be/eng/home_eng.asp>

# **Bulgaria** (Draft) National Influenza Pandemic Preparedness plan of the republic of Bulgaria;

<http://www.mh.government.bg/> (Bulgarian; English translation available in non-public domain)

**Canada** (December 2006) Canadian Pandemic Influenza Plan *for the health sector* ;

<http://www.phac-aspc.gc.ca/cpip-pclcpi/index.html>

**Cyprus** Summary of the Influenza Pandemic Preparedness Plan;

<http://www.moh.gov.cy/MOH/moh.nsf/All/9914FB742F8A35D84225718700530986?OpenDocument>

**Czech Republic** (April 2004) The National Pandemic Plan of the Czech Republic;

<http://www.who.int/csr/disease/influenza/czechplan.pdf>; (November 2004) Act of the Government of the Czech Republic (No. 1107) [http://www.eiss.org/documentseiss_pandemic_plan_czech_republic.pdf](http://www.eiss.org/documents/eiss_pandemic_plan_czech_republic.pdf)

**Denmark** (April 2006) Beredskab for pandemisk influenza. National strategi of rammer for planaegning I sundhedsberedskabet; <http://www.sst.dk/publ/Publ2006/CFF/Pandemibered/pandemiplan2006.pdf>; Bilag til beredskab for pandemisk influenza <http://www.sst.dk/publ/Publ2006/CFF/Pandemibered/bilag_pandemiplan2006.pdf>

**Estonia** (November 2005) Influenza Pandemic Preparedness Plan

[http://www.sm.ee/est/HtmlPages/Influensapandemicpreparednessplan/$file/InfluenzaPandPrepPlanENG%20December%202005.doc](http://www.sm.ee/est/HtmlPages/Influensapandemicpreparednessplan/$file/InfluenzaPandPrepPlanENG December 2005.doc)

**Finland** (2006) Finnish national preparedness plan for an influenza pandemid. Proposal of the Working Group on National Pandemic Preparedness. <http://www.stm.fi/Resource.phx/vastt/tervh/lintuflunssa/kieliversiot.htx.i753.pdf>

**France** (January 2007) Governmental Plan for the prevention and control of `Influenza pandemic’:

http://www.grippeaviaire.gouv.fr/IMG/pdf/plan_national_version_anglaise.pdf

**Germany** (May 2007) Nationaler Influenza Pandemie Plan Teil 1-3:

<http://www.rki.de/cln_049/nn_200120/DE/Content/InfAZ/I/Influenza/Influenzapandemieplan.html>;

**Greece** (October 2005) National Influenza Pandemi Plan: <http://www.keel.org.gr/keelpno/National_plan.pdf>;

**Hungary** (October 2005) National Influenza Pandemic Preparedness plan: <http://www.eum.hu/index.php?akt_menu=2652&hir_reszlet=8>;

**Ireland** (Draft, January 2007) A model Plan for Influenza Pandemic Preparedness :

<http://www.ndsc.ie/hpsc/A-Z/EmergencyPlanning/AvianPandemicInfluenza/Guidance/PandemicInfluenzaPreparednessforIreland/>;

**Italy** National plan for preparedness and response to an influenza pandemic

<http://www.who.int/csr/disease/influenza/italianpandemicplan_english.pdf>

**Japan** (November 2005) Pandemic Influenza Preparedness Action Plan of the Japanese Government <http://www.mhlw.go.jp/english/topics/influenza/dl/pandemic02.pdf>

**Latvia** National Influenza Pandemic Plan (July 2006)

<http://phoebe.vm.gov.lv/misc_db/web.nsf/bf25ab0f47ba5dd785256499006b15a4/17cb8c1218bf81cdc2257313001f391a/$FILE/informativais_zinjojums_gripa_last.pdf>

**Lithuania** (2005) Influenza Pandemic Preparedness Plan

[http://www.vvspt.lt/aktai/gripas/2005%2009%2020%20GRIPO%20PLANO%20VERT.pdf](http://www.vvspt.lt/aktai/gripas/2005 09 20 GRIPO PLANO VERT.pdf)

**Luxemburg** (2006) Plan gouvernemental Pandémie grippale <http://www.grippeaviaire.public.lu/mesures/gouv/plan_gouv_pandemie_grippale.pdf>

**Malta** no plan available on public domain

**Mexico** (December 2006) Plan nacional de preparación y respuesta ante una Pandemia de Influenza <http://www.dgepi.salud.gob.mx/pandemia/FLU-aviar-PNPRAPI.htm>

**Poland** The National Influenza Pandemic Preparedness plan for Poland:

[http://www.fao.org/docs/eims/upload//221487/national_plan_ai_pol_en.pdf](http://www.fao.org/docs/eims/upload/221487/national_plan_ai_pol_en.pdf)

**Portugal** (January 2006) Plano de contingência nacional para a pandemia de gripe.

<http://www.dgs.pt/upload/membro.id/ficheiros/i007770.pdf>

**Romania** National Influenza Pandemic Plan <http://www.ms.ro/>

**Slovak Republic** (November 2005) Detailed plan of measures in case of an influenza pandemic in the Slovak Republic: <http://www.health.gov.sk/redsys/rsi.nsf/0/D2869A65B5F83280C12570EC00517352?OpenDocument>;

**Slovenia** (July 2006**)** Načrt pripravljenosti na pandemijo gripe na področju zdravstva <http://www.mz.gov.si/si/delovna_podrocja/javno_zdravje/>

**Spain** (December 2006) National Pandemic Influenza Preparedness and response plan updata

<http://www.msc.es/ciudadanos/enfLesiones/enfTransmisibles/docs/ActualizacionPlanDic2006v_ing.pdf>

**Sweden** (November 2006) Influenza Strategier för prevention och kontroll

<http://www.socialstyrelsen.se/Publicerat/2006/9367/2006-131-35.htm> (November2006)

(February 2007) Contingency planning for an influenza pandemic-National Measures

<http://www.socialstyrelsen.se/NR/rdonlyres/A3964D22-0A81-41E6-A08C-3E29C1CE0619/7016/20071304.pdf>

**The Netherlands** (November 2006) Operationeel deeldraaiboek 3. Bestrijding influenzapandemie:

<http://www.rivm.nl/cib/infectieziekten/Influenza/influenza_draaiboek.jsp>

**United Kingdom** (Draft March 2007) UK’s Health Departments´ Influenza Pandemic Contingency Plan,

<http://www.dh.gov.uk/en/PandemicFlu/index.htm>

**United States** (November 2005) Human Health Services Pandemic Influenza Plan <http://www.hhs.gov/pandemicflu/plan/pdf/HHSPandemicInfluenzaPlan.pdf>; Appendix D NVAC/ACIP Recommendations for Prioritization of Pandemic Influenza Vaccine and NVAC Recommendations on Pandemic Antiviral Use <http://www.hhs.gov/pandemicflu/plan/appendixd.html>

Department of Health and Human Services, Pandemic planning update 4 (2007) <http://www.pandemicflu.gov/plan/panflureport4.pdf>
